# Total glycosides from *Eucommia ulmoides* seed promoted osteogenic differentiation of adipose-derived mesenchymal stem cells and bone formation in ovariectomized rats through regulating Notch signaling pathway

**DOI:** 10.1186/s13018-021-02797-5

**Published:** 2021-11-06

**Authors:** Yu-hu Zhou, Qiang Xie

**Affiliations:** 1 Department of Orthopedics, Yan’an University Affiliated Hospital, Yan’an, 716000 Shaanxi Province China; 2 Department of Orthopedics, Tianshui Combine Traditional Chinese and Western Medicine Hospital, No. 26, Weibingbei Road, Maiji District, Tianshui, 741020 Gansu China

**Keywords:** Total glycosides from *Eucommia ulmoides* seed, Adipose‐derived mesenchymal stem cells, Osteogenic differentiation

## Abstract

**Background:**

Osteoporosis (OP) is a well-known chronic degenerative disease, with impaired mesenchymal stem cells (MSCs) function and suppressed osteogenic differentiation. Total glycosides from Eucommia ulmoides seed (TGEUS) was a Chinese medicine and have rich pharmacological effects. This study was designed to explore the mechanism of TGEUS in promoting osteogenic differentiation and bone formation in ovariectomized (OVX) rats.

**Methods:**

Adipose‐derived mesenchymal stem cells (ADSCs) were isolated and treated with different concentration of TGEUS. Cell viability was assessed using cell counting kit-8 (CCK-8) assay. Osteogenic capacity was identified by ALP staining and ARS staining. Moreover, RNA sequencing between control and TGEUS treated ADSCs were further performed to reveal the mechanism of TGEUS in promoting osteogenic differentiation. The expression of Jag1, Lfng and Hey1 were measured using quantitative real-time polymerase chain reaction (qRTPCR). Osteogenic markers were further assessed by western blot. DAPT and NICD were further used to identify whether Notch signaling pathway involved into TGEUS promoting osteogenic differentiation of ADSCs. Ovariectomy-induced bone loss rats model was established and divided into three groups: sham, OVX and OVX + TGEUS groups. HE staining and immunohistochemical staining were further performed to identify whether TGEUS could promote bone formation.

**Results:**

TGEUS treatment significantly enhanced the cell viability and ALP activity than control group, the optimal dose of TGEUS was 5 μM. We selected 5 μM TGEUS for further study. TGEUS significantly enhanced ALP activity and calcium deposition than that of control group. Activation of Notch signaling fully blocked TGEUS-induced osteogenic differentiation of ADSCs. Following TGEUS treatment, the trabecular bone of the rats was significantly increased, thickened, and more connected compared to the OVX group. With the treatment of TGEUS, the expression of Osterix (Osx), Osteocalcin (OCN) and RUNX Family Transcription Factor 2 (RUNX2) increased than OVX group.

**Conclusion:**

TGEUS enhanced osteogenic differentiation of ADSCs and promoted bone formation in ovariectomy-induced bone loss rats. Our study broadened the understanding of TGEUS as a therapeutic target against osteoporosis.

## Background

Osteoporosis is characterized by low bone-mass density and microarchitectural deterioration of bone tissue [1, 2]. The incidence of osteoporosis in postmenopausal women is 30–55% [3]. Postmenopausal osteoporosis is attributed to excess bone resorption due to an estrogen deficiency [4, 5]. Although many drugs can be used for treatment of osteoporosis, its use remains controversial due to its efficacy and safety [6, 7]. Other anti-osteoporosis drugs (e.g., bisphosphonates and calcitonin) function as inhibitors of bone resorption, but their ability to increase or recover bone mass is minimal [8–10]. To restore the extensive bone loss, there is a great urgent to explore new drugs for building new bone [11]. Therefore, it is very popularity to seek natural alternatives for treatment of osteoporosis [12–14].

*Eucommia ulmoides* Oliv., also called Du‐Zhong in China, is one of the oldest and most important tonic herbs in traditional Chinese medicine (TCM) [15]. Additionally, Du-Zhong is also a popular folk drink and is used as a functional food preventing miscarriages and improving liver and kidney tone [16]. Du Zhong cortex extract (DZCE) has been used to protect bone loss in osteoporosis rats model [17].

Total glycosides from *Eucommia ulmoides* seed (TGEUS) was extracted from Du‐Zhong. Previous study revealed that TGEUS could improve the bone density in rats [16]. However, its mechanism of action is still unclear. Notch signaling pathway plays a crucial role in bone formation and bone remodeling. Notch signaling is a highly conserved pathway that primarily regulates osteogenic differentiation of stem cells. However, whether TGEUS could regulate Notch signaling pathway was unknown. In this study, we proved that TGEUS promoted osteogenic differentiation of ADSCs through regulating Notch signaling pathway.

## Material and methods

### Chemical reagents

Total glycosides from Eucommia ulmoides seed (Purity: 99.0%) was obtained from the Jiahe Duzhong Industry Co. Ltd (Lueyang, China). TGEUS was dissolved in DMSO and the final concentration of DMSO in the culture media remained below 0.05%.

### NICD overexpression and DAPT treatment

The Notch 1 Intracellular Domain (NICD) overexpression vector (3× Flag-NICD1) was purchased Suzhou Jima Gene Co. Ltd. (Suzhou, China). In brief, ADSCs were incubated into the 6-well plate and then transfected with 3× Flag-NICD1 plasmids using Lipofectamine 3000 to activate Notch signaling pathway. *N*-[*N*-(3,5-difluorophenacetyl)-*L*-alanyl]-*S*-phenylglycine *t*-butyl ester (DAPT) was an inhibitor of Notch signaling pathway and was purchased from MedChemExpress (Shanghai, China). The concentration of DAPT was 10 μM.

### Isolation of human ADSCs

Human adipose tissue was harvested from the subcutaneous fat (1 cm^3^) of the abdominal wall during gynecologic surgery. Informed consent Informed consent was obtained from all participants. Human adipose tissue was rinsed for 3 times with PBS to remove visible fascia and red particles cells. Then, an equal volume of 0.075% type I collagenase was added and digested for 30–45 min at 37 °C. An equal volume of DMEM/F12 medium containing 10% fetal bovine serum (FBS) was added to stop the digestion, and centrifuged at 15000 rpm for 10 min. Next, 5 mL red blood cell lysate was added and resuspend at room temperature for 5 min. After filtering through a 70 μm cell sieve, all cells were transferred to culture dish cell culture.

### Osteogenic differentiation of ADSCs

For osteoblastic differentiation, after reaching 80% confluence, ADSCs were cultured in alpha-MEM supplemented with 10% FBS, 10 nM dexamethasone, 0.2 mM L-ascorbic acid, and 10 mM β-glycerophosphate for 7 or 21 days. The medium was changed daily for 2 days. The well-grown ADSCs from the third generation were randomly divided into three groups. The cells of the induce group were induced into osteoblasts by osteogenic induction medium. TGEUS group was additional added TGEUS (5 μM) in osteogenic induction medium. Another two groups were NICD and NICD + TGEUS groups.

### RNA sequencing analysis

ADSCs were treated with vehicle or TGEUS (5 μM) for 48 h. Total RNA was isolated from ADSCs using TRIzol method. RNA sequencing was performed by OEbiotech Company (Shanghai, China). Differentially expressed genes were identified via the “limma” R package. The selection criteria were *p* adjust < 0.05 and |log2 (fold change, FC)|≥ 1. Heatmap of the differentially expressed genes were selected by R software with heatmap package. Gene ontology (GO) analysis and Kyoto Encyclopedia of Genes and Genomes (KEGG) pathway enrichment analysis performed on the Database of Annotation Visualization and Integrated Discovery (https://david.ncifcrf.gov/). Moreover, the enrichment figures were drawn by R software.

### Reverse transcription-polymerase chain reaction (qRT-PCR)

The bone samples were mortared into powder under sterile conditions. Samples then were transferred to 1.5-mL Eppendorf centrifuge tubes. Total RNA was extracted using Trizol (Invitrogen) according to the manufacturer’s instruction. cDNA was prepared using the PrimeScript RT reagent Kit with gDNA Eraser (Takara). The RNA concentration was measured with a NanoDrop 2000 (Thermo Fisher Scientific). qRT-PCR was performed using SYBR Premix Ex Taq II (Takara) in CFX96 Real-Time System (Bio-Rad). Relative gene expression was normalized by GAPDH using a 2^−ΔΔCt^ method. The primers were synthesized by Gene Script Company (Nanjing, China). The results of each group are presented as mean ± SD.

### Western blotting assay

Protein was isolated with radioimmunoprecipitation assay buffer (RIPA) lysis buffer (Sangon Biotech Co, Ltd) containing 1 mmol/l phenylmethanesulfonyl fluoride on ice. Subsequently, the cell lysates were centrifuged at 15,000*g* at 4 °C for 10 min. The samples were heated at 95 °C for 5 min in sample buffer containing 2% SDS and 1% 2- mercaptoethanol, lysates were separated using 10–12% SDS-PAGE and electro-transferred onto a polyvinylidene fluoride membrane (PVDF, Millipore, USA). The PVDF with proteins were then blocked with 5% (w/v) nonfat milk for 1.5 h to block the non-specific sites on blots. The primary antibodies dissolved in the blocking buffer were used to determine the targeting protein blots overnight at 4 °C. After rinsing, the nitrocellulose membranes were incubated with Goat Anti-Rabbit IgG H&L (Alexa Fluor® 488) as a secondary antibody for 1 h incubation at room temperature. Primary antibodies, including OSX, OCN and RUNX2 and GAPDH were purchased from Proteintech (Wuhan, Hubei province, China) and used according to manufacturer’s instructions. Immunoreactive bands were visualized by the Odyssey Infrared Imaging System.

### CCK-8 assay

An CCK-8 assay was used to assess ADSCs proliferation. The cells were seeded in a 96-well plate at a density of 2000 cells/well and then incubated at 37 °C and 5% CO_2_. CCK-8 solution (500 μg/ml) was added to each well and incubated for 3 h. Formazan crystals were dissolved in DMSO and the intensity of the CCK-8 product was measured at 540 nm using a microplate reader (FlexStation 3, Molecular Devices, USA) to determine the OD value of each well after 30 min.

### Alkaline phosphatase activity assay

To quantify osteogenic differentiation, an ALP assay, which is used as an early marker of osteogenic differentiation, was performed. Cells were seeded in a six-well plate at a density of 1 × 10^5^ cells/ml and cultured in osteogenic medium. An amount of 50 μl of the supernatant was transferred into a 96-wells plate and 150 μl of alkaline phosphatase kit (Sigma Aldrich, USA) was added to it in the ratio of 4 R1/R2 to 1. Finally, the alkaline phosphatase activity was measured using the microplate reader (FlexStation 3, Molecular Devices, USA) at a wavelength of 450 nm.

### Alkaline phosphatase (ALP) and Alizarin red S (ARS) staining

ALP and ARS staining was performed for to identify the early and late osteogenic capacity respectively. Briefly, 12-well-plate were washed with PBS for three times and then fixed with 4% paraformaldehyde for 10 min. Then, ADSCs were stained with the ALP buffer staining solution (Solarbio, Beijing, China) for 30 min. Then, the stained cells in each well were photographed under the standard light microscopy (Nikon ECLIPSE TS100). The staining was quantified using Image-Pro Plus 6.0 (Media Cybernetics, USA). For ARS staining, ADSCs were fixed with 4% paraformaldehyde for 10 min. And ADSCs were incubated with ARS solution (2%, Solarbio, Beijing, China) for 30 min. To quantify Alizarin Red S-retained minerals, 10% (v/w) cetylpyridinium chloride (Carl Roth, Karlsruhe, Germany) at room temperature for 1 h and the Alizarin Red stain in extraction buffer was determined by measuring the optical density (OD) of the solution at 560 nm.

### Ovariectomy (OVX) induced bone loss model

A total of 60 rats were randomly divided into three groups: sham, OVX and OVX + TGEUS groups. All rats were anesthetized intraperitoneal with 10% chloral hydrate (0.2 mL/100 g) to reach satisfactory anesthesia. In the sham group, only about 1 g of adipose tissue around the ovaries was removed, and the ovaries were retained. For OVX group, the ovaries were removed by bilateral excision. After flushed with saline, and the wound was sutured layer by layer, followed by disinfection of the skin. Then, for OVX + TGEUS group, TGEUS (400 mg/kg body weight/day) was subcutaneously injected according to previously report [18].

### HE and immunohistochemical staining

For HE-staining, the sections were stained using standard procedures. After paraffin embedding, embedded tissues were sliced to 5 μm. After drying at 60 °C for 60 min, the slides were deparaffinized. Slides were incubated in hematoxylin solution for 6 min, rinsed in water, and then incubated in eosin solution for 2 min. After the sections were sealed with neutral balsam, photos were taken and analyzed.

For immunohistochemical staining, 0.01M citric acid repair liquid (pH 6.0) was added to repair antigen under high pressure. Then, slides were added primary antibody rabbit anti-OSX, OCN and RUNX2. Immunohistochemistry (IHC) was carried out in strict accordance with the second antibody kit instructions. Sections were observed under an Olympus Optical AX70 microscope (Olympus, Tokyo, Japan) and photos were taken.

### Statistical analysis

Statistical analysis Statistical analysis was performed using GraphPad Prism software (version 7; GraphPad Software, Inc.). One-way analysis of variance (one-way ANOVA) with Bonferroni's post hoc test for normally distributed data and Kruskal–Wallis nonparametric test for skewed data were used to compare three or more means. For all analyses, differences with *p* < 0.05 were considered statistically significant.

## Results

### TGEUS enhanced osteogenic differentiation of ADSCs

**Fig. 1:**
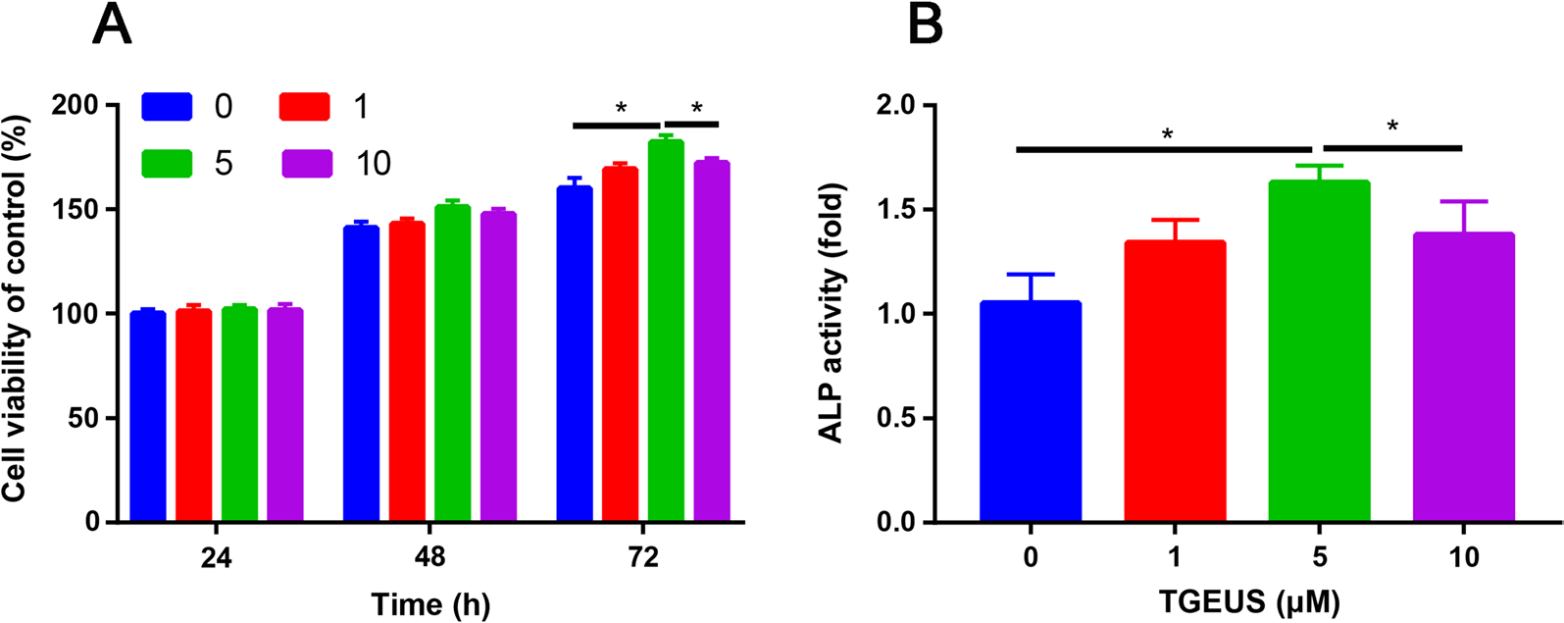
**A** Cell viability of ADSCs in different concentration of TGEUS (0, 1, 5 and 10 μM) at 24, 48 and 72 h; **B** ALP activity of ADSCs in different concentration of TGEUS (0, 1, 5 and 10 μM). *P < 0.05

To explore the optimal concentration of TGEUS for osteogenesis, cell viability assay and ALP activity results were all taken into consideration. TGEUS treatment significantly enhanced the cell viability (Fig. 1A) and ALP activity (Fig. 1B) than control group, the most prominent concentration was 5 μM TGEUS. However, cell viability significantly declined when the concentration exceeded 5 μM (Fig. 1 A and B). The optimal concentration of TGEUS-treated was determined to be 5 μM based on ALP activity and cell viability and thus chosen for subsequent experiments.

### RNA sequencing between control and TGEUS-treated ADSCs

**Fig. 2:**
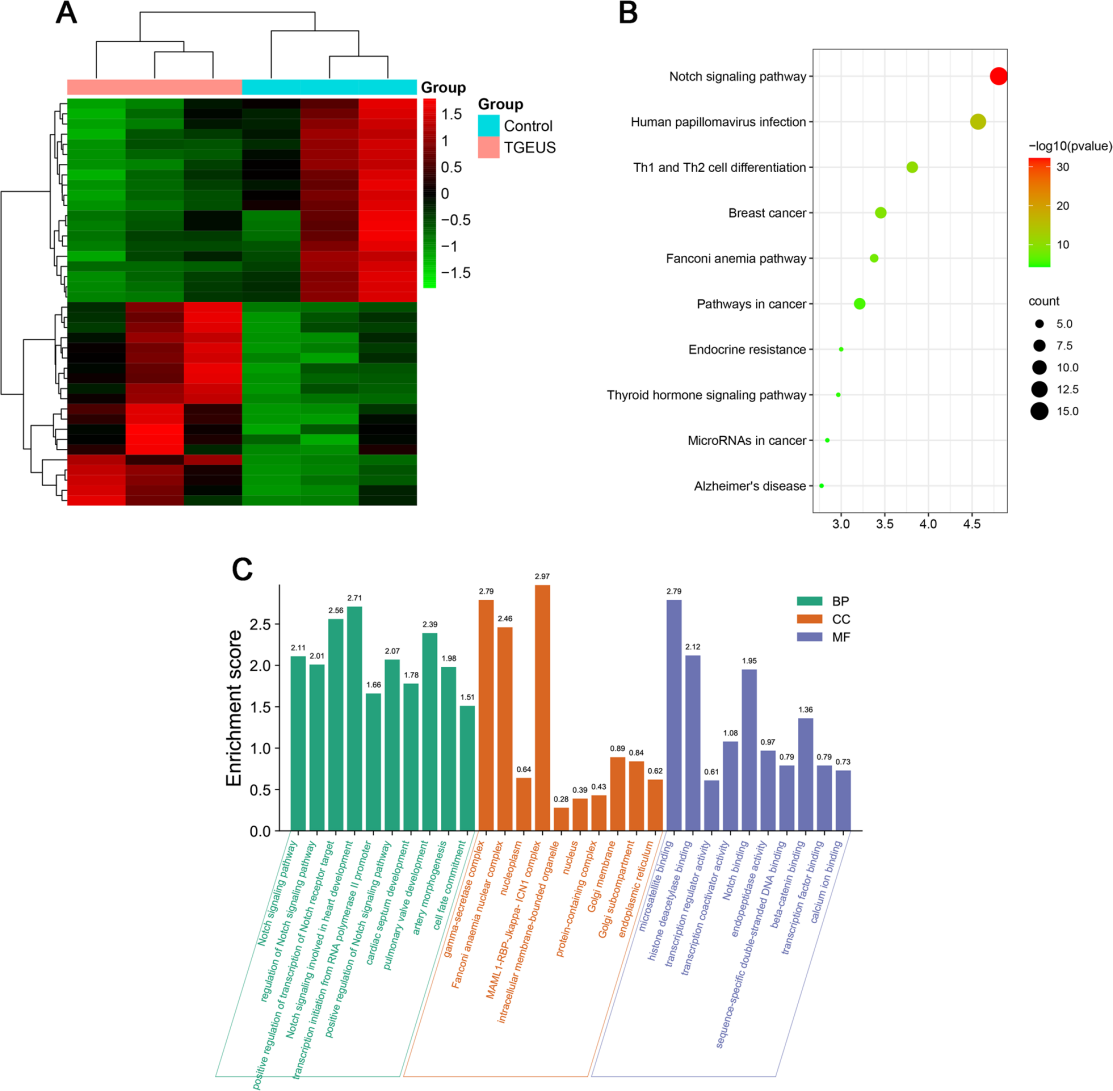
**A** Heatmap of the top 100 differentially expressed genes between control and TGEUS treated ADSCs. **B** KEGG pathway analysis of the differentially expressed genes between control and TGEUS treated ADSCs. **C** Gene ontology (biological process, cellular component and molecular function) of the differentially expressed genes

To understand the molecular mechanism by which TGEUS promotes osteoblast differentiation of ADSCs, we performed RNA-sequencing analysis and comparing TGEUS-treated and DMSO-treated control cells, during osteoblast differentiation. A total of 355 differentially expressed genes were identified, of which 270 were upregulated, and 185 were downregulated. Top 50 differentially expressed genes between TGEUS and normal ADSCs were shown in Fig. 2A.

Top ten enrichment terms of biological process mainly including: single-organism metabolic process, cellular response to stress, phosphate-containing compound metabolic process, cellular metabolic process, phosphorus metabolic process, nucleobase-containing compound catabolic process, cellular response to stimulus, positive regulation of protein metabolic process, regulation of protein metabolic process and aromatic compound catabolic process (Fig. 2B).

Top ten enrichment terms of cellular component mainly including: cytosol, intracellular organelle lumen, membrane-enclosed lumen, organelle lumen, vesicle, membrane-bounded vesicle, anchoring junction, adherens junction, extracellular organelle and extracellular membrane-bounded organelle (Fig. 2B).

Top ten enrichment terms of molecular function mainly including: poly(A) RNA binding, RNA binding, protein phosphatase binding, protein binding, S100 protein binding, phosphatase binding, ubiquitin protein ligase binding, small conjugating protein ligase binding, enzyme binding and structural constituent of muscle (Fig. 2B).

After KEGG pathway enrichment analysis, the differentially expressed genes (DEGs) were mainly enriched in 10 KEGG pathways including Notch signaling pathway, Human papillomavirus infection, Th1 and Th2 cell differentiation, Breast cancer, Fanconi anemia pathway, Pathways in cancer, Endocrine resistance, Thyroid hormone signaling pathway, MicroRNAs in cancer and Alzheimer's disease (Fig. 2C).

**Fig. 3:**
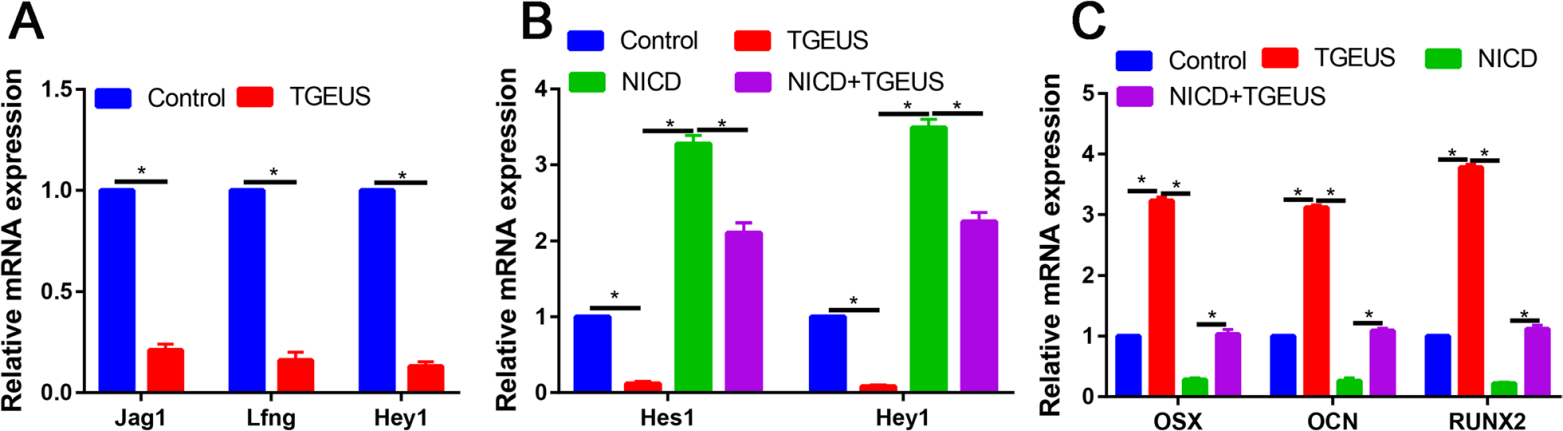
**A** Relative Jag1, Lfng and Hey1 expression in control and TGEUS treated ADSCs. **B** Relative Hes1 and Hey1 expression in control, TGEUS, NICD and NICD + TGEUS groups. **C** Relative OSX, OCN and RUNX2 expression in control, TGEUS, NICD and NICD + TGEUS groups

In accordance with the RNA sequencing results, TGEUS significantly increased the expression levels of Jag1, Lfng and Hey1 than control group, the difference was statistically significant (Fig. 3A and B).

**Fig. 4:**
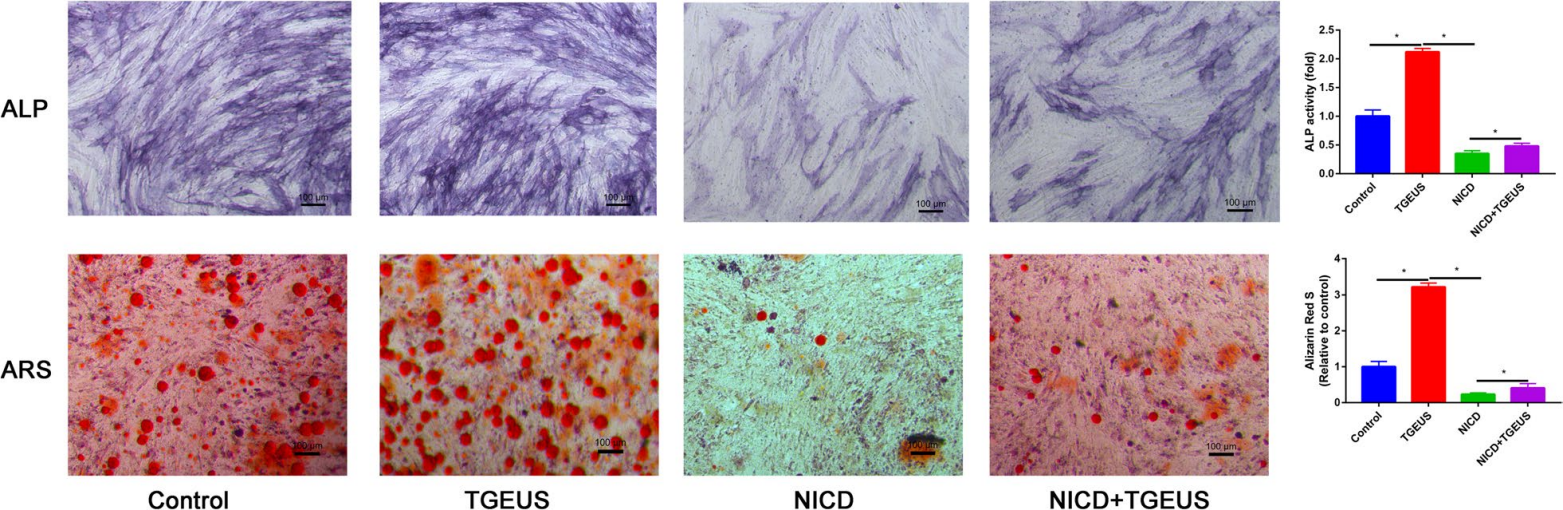
ALP and ARS staining in control, TGEUS, NICD and NICD + TGEUS groups

ALP staining and ARS staining confirmed that TGEUS treatment promoted osteoblast differentiation (Fig. 4). NICD significantly downregulated ALP activity and calcium deposition than control groups (Fig. 4). What’s more, NICD partially blocked the effects of TGEUS treatment promoted osteoblast differentiation of ADSCs (Fig. 4).

We finally assessed the osteogenic markers (OSX, OCN and RUNX2) in control, TGEUS, NICD and NICD + TGEUS groups. We found that TGEUS significantly enhanced the expression of osteogenic markers. NICD significantly blocked these genes expression. Moreover, NICD partially reversed the promotion effects of TGEUS in promoting osteogenic differentiation of ADSCs.

**Fig. 5:**
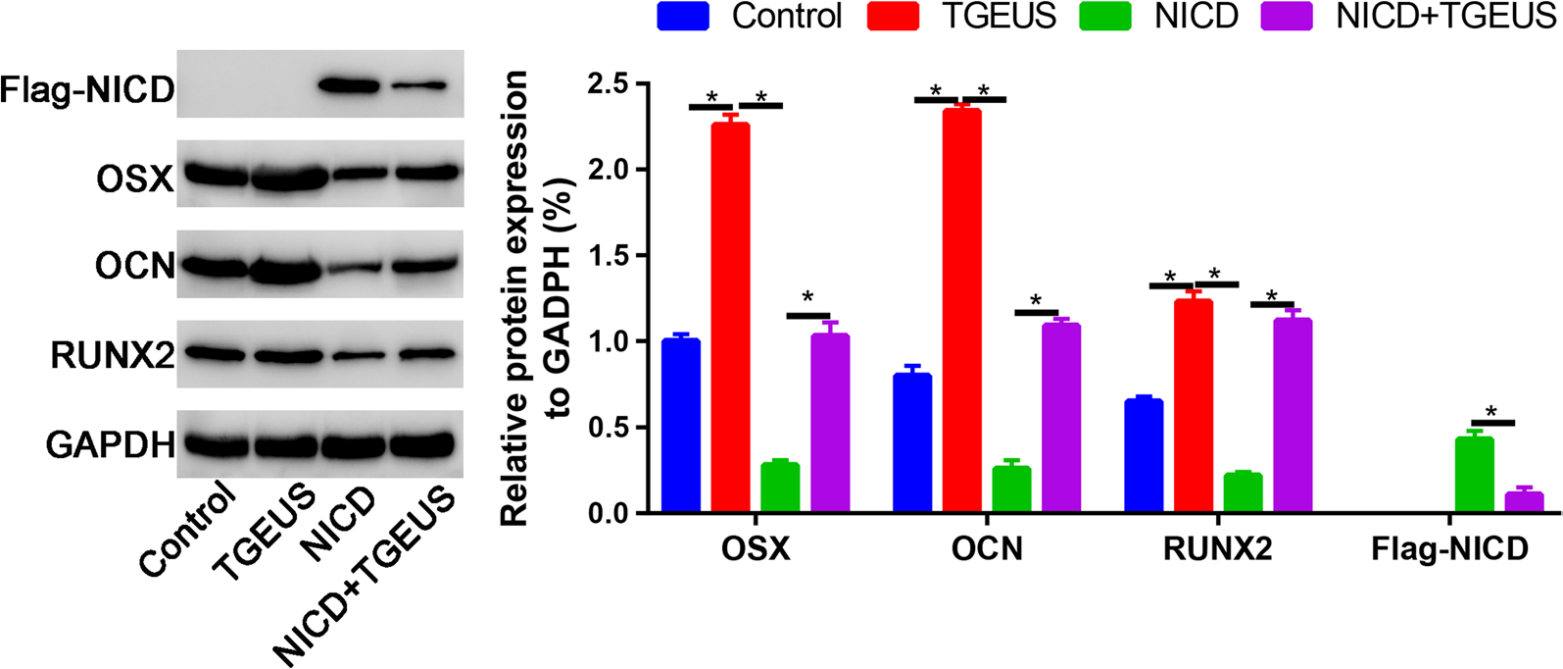
Relative Flag-NICD, OSX, OCN and RUNX2 expression in control, TGEUS, NICD and NICD + TGEUS groups

Western blot analysis was in agreement with the quantitative real-time PCR (qRT-PCR) results, showing that the protein expression of OSX, OCN and RUNX2 was up-regulated in the TGEUS group (Fig. 5). This promotion effect was partially blocked by NICD overexpression vector, which suggested that activating Notch signaling pathway partially reversed the promoting effects of TGEUS.

### DAPT enhances TGEUS-induced osteogenic differentiation

**Fig. 6:**
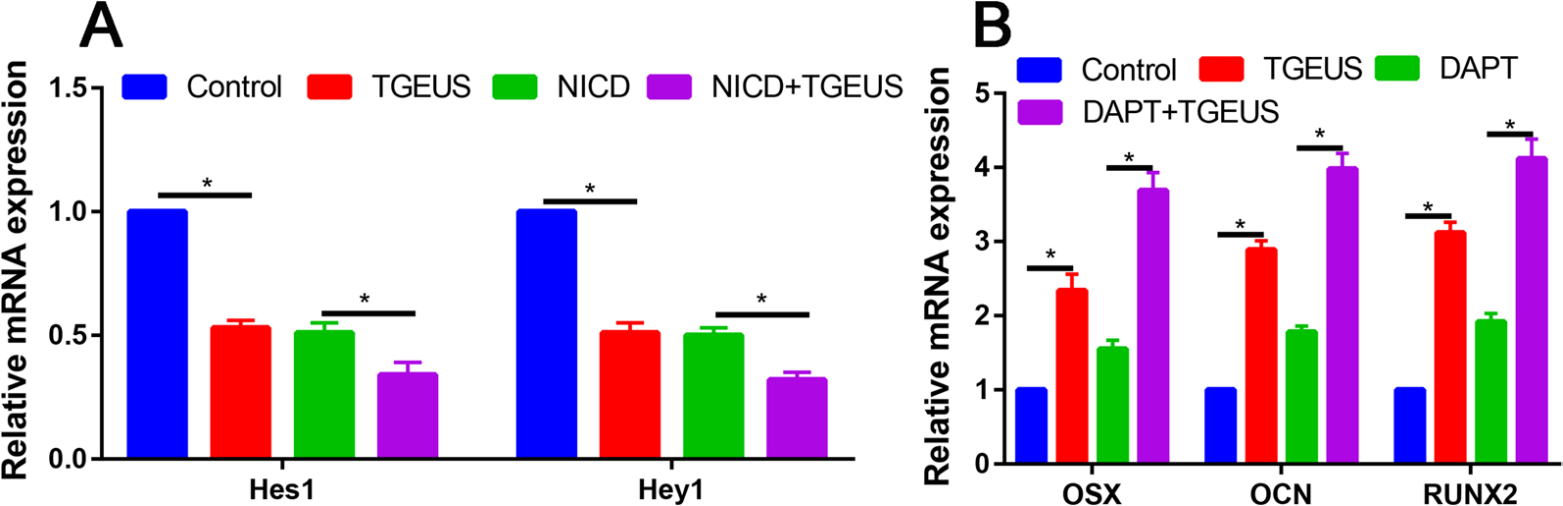
**A** Relative Hes1 and Hey1 mRNA expression in control, TGEUS, NICD and NICD + TGEUS groups. **B** Relative OSX, OCN and RUNX2 expressions in control, TGEUS, DAPT and DAPT + TGEUS groups

**Fig. 7:**
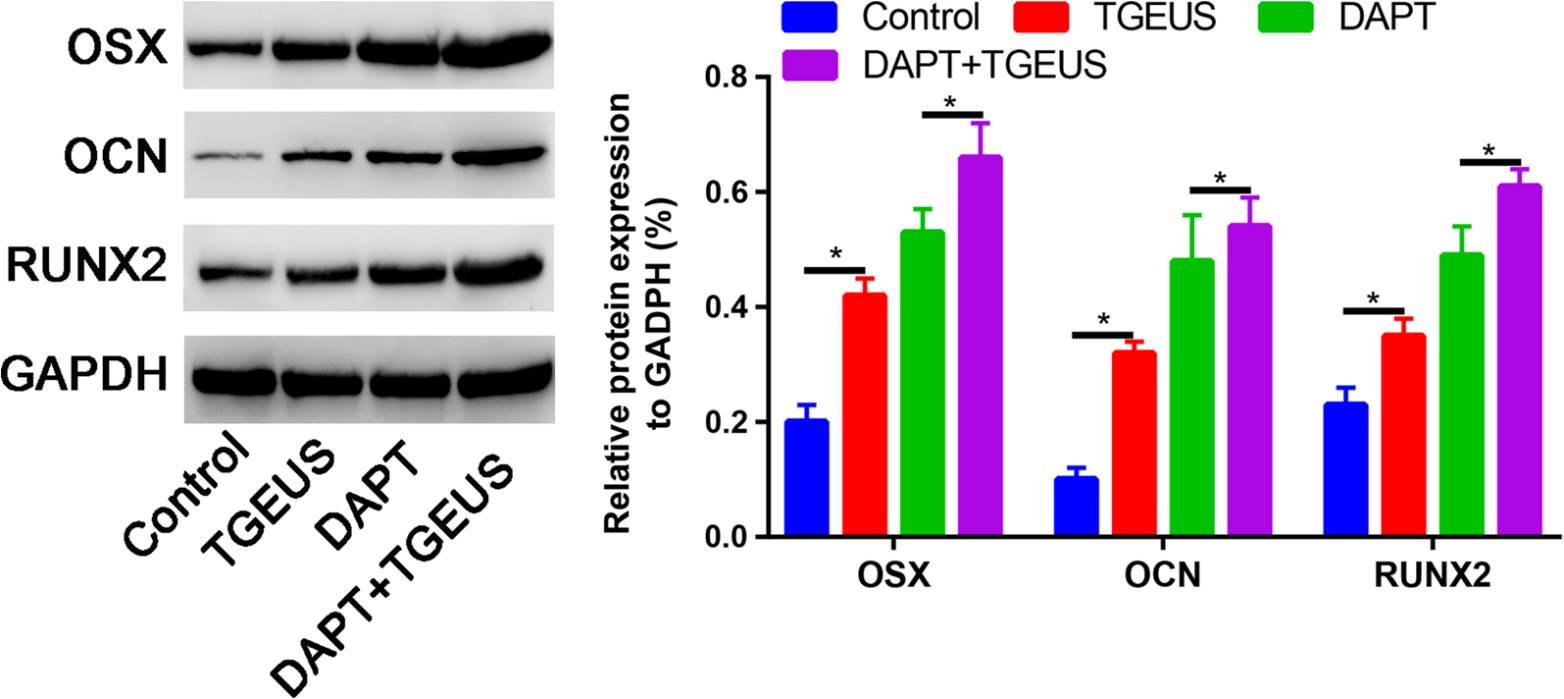
Relative OSX, OCN and RUNX2 expression in control, TGEUS, DAPT and DAPT + TGEUS groups

NICD treatment decreased the Hes1 and Hey 1 expression than control group (Fig. 6A). DAPT treatment reduced the mRNA expression levels of Hes1 and Hey1 than control group (*P* < 0.05, Fig. 6B). TGEUS combined DAPT significantly increased the OSX, OCN and RUNX2 expression than TGEUS or DAPT alone in osteogenic induction ADSCs (Fig. 7).

### TGEUS treatment prevents OVX-induced bone loss by inhibiting Notch signaling

**Fig. 8:**
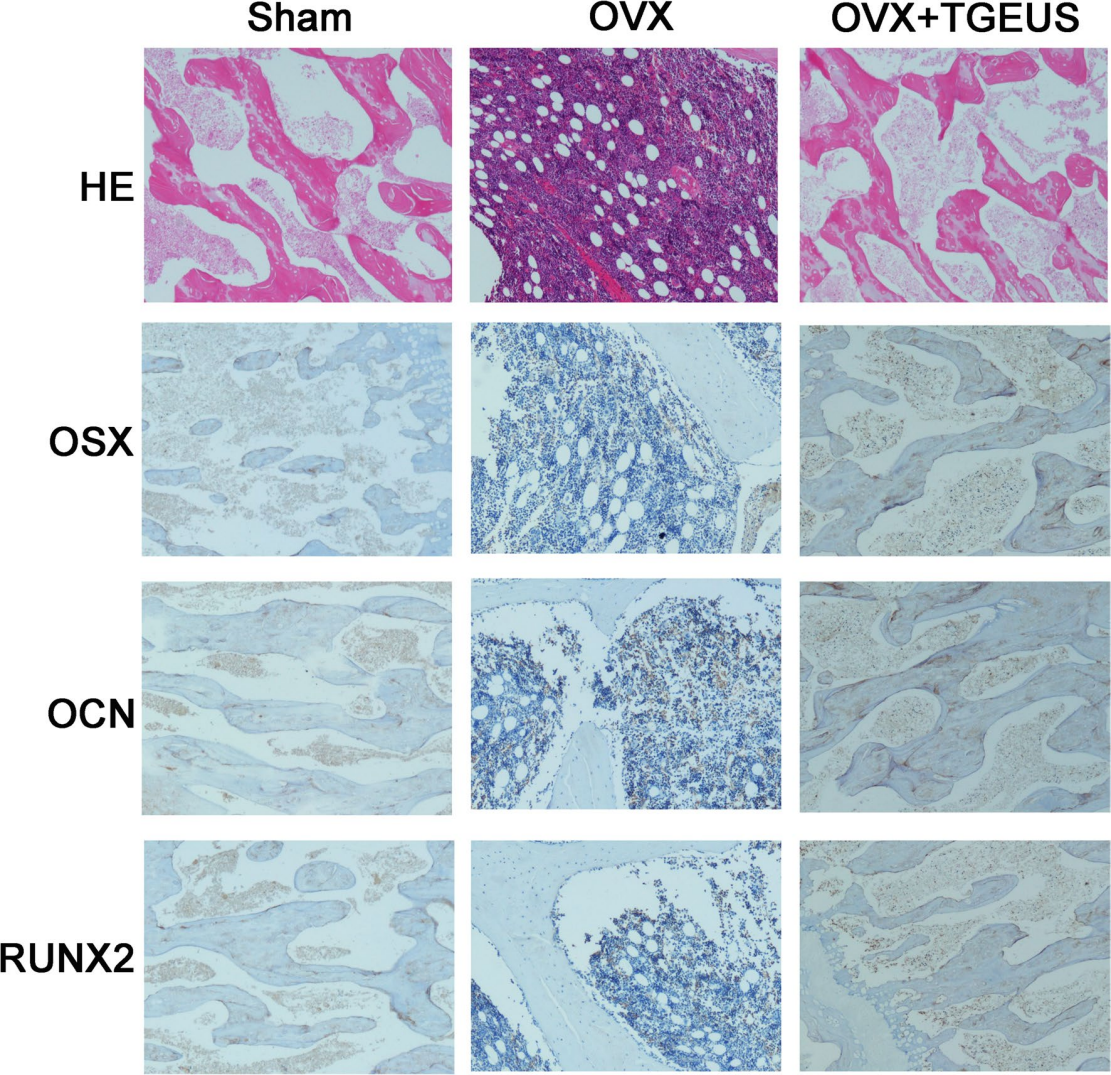
HE staining and immunohistochemical staining for OSX, OCN and RUNX2 in sham, OVX and OVX + TGEUS groups

In order to assess TGEUS -induced changes in the bone microstructure, the bone tissues of distal femurs were carefully harvested and scanned via HE staining at 3 months after ovaries removed (Fig. 8).

Compared with sham group, ovaries removed revealed fewer and much thinner trabeculae, which indicated that the model was successfully established (Fig. 8).

Following treatment with TGEUS, the femoral trabeculae were greatly improved compared to the OVX group (Fig. 8).

The protein expression of osteogenic markers (OSX, OCN and RUNX2) were measured by immunohistochemical staining. OVX dramatically decreased the OSX, OCN and RUNX2 expression than that of sham group, and these tendencies could be partially reversed by treatment with TGEUS (Fig. 8).

## Discussion

In this study, we found that TGEUS promoted osteogenic differentiation of ADSCs through inhibiting Notch signaling pathway. Moreover, TGEUS prevented bone loss in ovariectomy-induced rats model. Both in vitro and in vivo model suggested that TGEUS is a potential anti‐osteoporotic therapeutic.

Osteoporosis is a chronic disease associated with decreased bone density that afflicts millions of people worldwide [19]. Traditional Chinese Medicines have been widely used in the prevention and treatment of post-menopausal osteoporosis [10, 20–22]. TGEUS can be developed as a potential anti-osteoporotic therapeutic [23]. However, the precise mechanisms underlying the beneficial effects of TGEUS in promoting osteogenesis are not fully understood. Therefore, we performed this study to further identify the mechanism of TGEUS in promoting bone formation.

The Notch signaling pathway plays a significant role in skeletal development, adult skeletal homeostasis, and bone remodeling [24, 25]. In the canonical Notch pathway, the single-transmembrane cell surface receptors undergo sequential proteolytic cleavage upon binding of their ligand [26]. Notch plays a critical role in skeletal development and homeostasis, and serious skeletal disorders can be attributed to alterations in Notch signaling [27]. When Notch is expressed in differentiated osteoblasts or in osteoblast precursors, it suppresses osteoblastic gene markers in vitro and causes osteopenia in vivo [28]. In this study, TGEUS significantly downregulated the expressions of Hes1 and Hey1. Hes1 and Hey1 expressions were two main molecules of Notch signaling pathway. Thus, TGEUS could directly regulate Notch signaling pathway. While administration with NICD could partially enhanced the osteogenic differentiation of ADSCs. ADSCs were widely used in orthopedics to promote bone regeneration [29].

RNA sequencing was performed to identify the mechanism of TGEUS in promoting osteogenic differentiation of ADSCs. Employing the loss- and gain-of-function strategies, we show that inhibiting Notch signaling pathway enhanced the TGEUS-induced osteogenic differentiation of ADSCs. TGEUS promoted osteogenic differentiation and calcification, as shown by the increased of osteogenic marker levels, including RUNX2, OSX and OCN. limitations of this study are as follows. (1) the receptor of TGEUS need for more studies to validate. (2) Specifically, the toxicity of our drugs in vivo requires further experiments.

## Conclusion

In conclusion, our data suggested that TGEUS promotes osteogenic differentiation of ADSCs and reverses bone loss induced by ovariectomy in rats. These effects were mainly through inhibition of the Notch signaling pathway. Therefore, we speculated that TGEUS inhibiting Notch signaling pathway and subsequent stimulate osteoblast differentiation and bone formation. Thus, our finding results suggest that TGEUS is a promising alternative treatment candidate for osteoporosis.

## Data Availability

All data supporting the findings are within the manuscript.

## Abbreviation

OP: Osteoporosis
MSCs: Mesenchymal stem cells
TGEUS: Total glycosides from Eucommia ulmoides seed
ADSCs: Adipose‐derived mesenchymal stem cells
qRTPCR: Quantitative real-time polymerase chain reaction
ET: Estrogen therapy
TCM: Traditional Chinese medicine
DZCE: Du Zhong cortex extract
NICD: Notch 1 Intracellular Domain
DAPT: N-[N-(3,5-difluorophenacetyl)-L-alanyl]-S-phenylglycine t-butyl ester
FBS: Fetal bovine serum
RIPA: Radioimmunoprecipitation assay buffer
PVDF: Polyvinylidene fluoride membrane
ALP: Alkaline phosphatase
ARS: Alizarin red S
OVX: Ovariectomy
IHC: Immunohistochemistry
OVX: One-way analysis of variance
